# The Value of the Wenker Rubber Jaw as an Aid in Teaching Therapeutics

**Published:** 1902-08

**Authors:** Harvey N. Jackson

**Affiliations:** Milwaukee, Wis.


### THE VALUE OF THE WENKER RUBBER JAW AS AN AID IN TEACHING THERAPEUTICS.[1]

BY HARVEY N. JACKSON, D.D.S., MILWAUKEE, WIS.

THE Wenker rubber jaw has been so helpful to me in my work with students, and the possibilities that suggest themselves seem so great as I continue in its use, that I feel that I should call the attention of teachers in this branch of dentistry to its advantages, not only as a means whereby the technic of the application of remedies can be nicely shown, but also as a means to keep the class interested, and as a rest and change from the ordinary manner of lecturing.

It may be advantageously used in teaching the removal of calculus, the different instruments used, and their manipulation in removing deposits in the most effectual way with the least discomfort to the patient; to illustrate the use of cups, brushes, and floss-silk in polishing the teeth; in the treatment of putrescent teeth, as to the shape of the cavity, the opening of the pulp-chamber, the exposure of the chamber floor, the mouths of the canals, and gaining free access thereto, followed by the actual employment of the medicinal agents, showing the use of broaches, canal reamers, etc., and, in fact, every step in the process of treating teeth can be demonstrated nearly as well as it can be in an actual clinic in the mouth; the application of a devitalizing agent; the ways and means of properly sealing the agent in the tooth to get the best results and prohibit its escape to the gum tissue; the different means employed to safely confine the agent in badly broken down teeth, as the use of cement, the application of rubber tubing, Kirk's method of enveloping the tooth with adhesive plaster, or the

---

[1] Read before the Southern Wisconsin Dental Society, May, 1902.

use of the celluloid matrix as a retention wall, and so on through the list; the manner of applying and operating with mummifying agents; the presentation of pulp exposures, with the different methods of capping; the use of the pressure method with cocaine for the painless extirpation of pulps.

The ordinary method of treating abscess with fistulous opening by pumping an agent through the opening, or forcing through with syringe and rubber stopper, can be illustrated fully as well, I believe, as at the chair-side. To this end I have made a slit through the alveolar wall in the rubber jaw, at a point where the fistula usually exhibits itself; then, reaming the canal in an incisor tooth and drilling a hole through the apical end, I passed a string through the tooth, then down through the socket, and out of the slit representing the fistula. The tooth was then set with thin cement and the string withdrawn before the cement had hardened, thus preserving the opening. The several modes of procedure for forcing medicaments through the fistula can now be nicely exhibited.

The reaming of canals with the stubbed Detroit broach for the treatment of improperly so-called blind abscess can also be shown to advantage.

Several jaws can be filled with extracted teeth and thus enable the instructor to place before the class a large number exhibiting the different forms and conditions as they come to us in actual practice, and all this can be exemplified with the teeth practically *in situ,* which I have found much better than using the single teeth out of their normal position.

A table clinic can be given as often as seems necessary during the course of lectures; it helps to keep the students interested in what many of them find to be a dry subject, and they will get a much better understanding of the subject from the lectures by its use.

But the chief benefit gained for the student lies in the fact that it enables him to use his knowledge of therapeutics in a practical and intelligent way when the time of need comes.

My experience with students in the office and in college classes has been quite sufficient to teach me this truth: that to teach a student the derivation of a drug, its medical properties, action, and therapeutic uses is *one* thing, and to teach that student *how* to use and when to use it at the chair-side is *quite* another.

Many of our graduates of to-day who have not had the good fortune of receiving instructions in a dental office from a competent preceptor are exceedingly lame in therapeutic technics.

I have a case in mind which will serve to illustrate. A graduate from a prominent school who had been practising for two years treated a putrescent incisor tooth for several months without any success. The putrescent condition was of short standing, and grew worse from the treatment. It became necessary to treat the tooth every day to keep the patient comfortable. If the dressing were sealed in the tooth the patient invariably suffered severely.

The young dentist applied to an older practitioner for information as to the cause of the difficulty. Upon questioning, it was learned that he had been using antiseptics that were irritating and easily dissipated.

The older practitioner advised him what to use, but after a short trial the young dentist gave up discouraged and brought the patient to the senior dentist for consultation. Upon removing the dressing the cotton was found so offensive that the odor remained in the office for a considerable time after the patient left. The canal was washed and rewashed with dioxygen until the mephitic gases were scarcely perceptible, and then dressed with eucalyptol and sealed.

The young dentist returned in a few days and stated that the patient had been very comfortable since that treatment. The technic of the treatment was a revelation to him. He learned what to use, but more particularly *how* to use it. He confessed that he had a great deal of trouble with putrescent teeth, and believed that he would progress nicely after seeing that demonstration.

And so I believe the use of the rubber jaw fits the student to go into the infirmary or into actual practice and apply the dental remedies with much more intelligence and effectiveness than he could without the use of it, or some substitute, to hold the teeth in a normal position.

In this little paper I have simply tried to give an idea of what can be accomplished with technic work employed collaterally with lectures. Many other ideas will present themselves to the thoughtful and ingenious worker in therapeutics who will employ this adjunct in his work as he continues in its use.

